# Transcription factor Runx1 is pro-neurogenic in adult hippocampal precursor cells

**DOI:** 10.1371/journal.pone.0190789

**Published:** 2018-01-11

**Authors:** Hirokazu Fukui, Annette Rünker, Klaus Fabel, Frank Buchholz, Gerd Kempermann

**Affiliations:** 1 German Center for Neurodegenerative Diseases (DZNE) Dresden, Dresden, Germany; 2 CRTD–Center for Regenerative Therapies Dresden, Technische Universität Dresden, Dresden, Germany; 3 Medical Systems Biology, Technische Universität Dresden, Dresden, Germany; Goethe-Universitat Frankfurt am Main, GERMANY

## Abstract

Transcription factor Runx1 (*Runt Related Transcription Factor 1*), plays an important role in the differentiation of hematopoetic stem cells, angiogenesis and the development of nociceptive neurons. These known functions have in common that they relate to lineage decisions. We thus asked whether such role might also be found for Runx1 in adult hippocampal neurogenesis as a process, in which such decisions have to be regulated lifelong. Runx1 shows a widespread low expression in the adult mouse brain, not particularly prominent in the hippocampus and the resident neural precursor cells. Isoforms 1 and 2 of Runx1 (but not 3 to 5) driven by the proximal promoter were expressed in hippocampal precursor cells ex vivo, albeit again at very low levels, and were markedly increased after stimulation with TGF-β1. Under differentiation conditions (withdrawal of growth factors) Runx1 became down-regulated. Overexpression of Runx1 in vitro reduced proliferation, increased survival of precursor cells by reducing apoptosis, and increased neuronal differentiation, while slightly reducing dendritic morphology and complexity. Transfection with dominant-negative Runx1 in hippocampal precursor cells in vitro did not result in differences in neurogenesis. Hippocampal expression of Runx1 correlated with adult neurogenesis (precursor cell proliferation) across BXD recombinant strains of mice and covarying transcripts enriched in the GO categories “neural precursor cell proliferation” and “neuron differentiation”. Runx1 is thus a plausible candidate gene to be involved in regulating initial differentiation-related steps of adult neurogenesis. It seems, however, that the relative contribution of Runx1 to such effect is complementary and will explain only small parts of the cell-autonomous pro-differentiation effect.

## Introduction

In a mouse genetic reference in panel adult hippocampal neurogenesis had a hereditability (h^2^) of 0.70 ± 0.05 but genetic association studies revealed no significant associations with large effect size [1]. The trait is highly polygenic and at the same time strongly and lastingly regulated by activity and environmental influences [2]. In such situation, analyzing the role of transcription factors, onto which regulatory programs might converge, is a promising strategy to identify the key elements of the complex control mechanisms.

Runx1 is an interesting candidate gene for several reasons. Runx (runt-related transcription factor) genes code for transcription factors that are critically involved in the transition between proliferation and differentiation [3], hence exactly matching the type of factor we were searching. Most of the relevant regulation comes from studies in hematology, where Runx1 mutations are causative for acute lymphatic leukemia (AML), hence the original name of the human gene: AML1. But a broader perspective on Runx genes in stem cell biology had already been developed [4]. A critical role in angiogenesis, for example, has been suggested [5].

Generally, the family of Runx genes appears to regulate stem cell quiescence, proliferation, and differentiation in a wide variety of cell types. Initial insight came from studies of *Caenorhabditis elegans* (*C*. *elegans*) [6]. A Runx1 ortholog in *C*. *elegans*, *rnt-1*, is predominantly expressed in stem cell-like seam cells. A *rnt-1* mutant was found to have impaired asymmetric division in seam cells.

Nevertheless, the participation of Runx1 in stem cell functions has been demonstrated in a wider range of systems. Intracellularly, Runx1 interacts with Smad transcription factors, which in turn are the transducers of transforming growth factor beta (Tgfb), bone morphogenic protein (BMP), and activin signal transduction [7].

In the nervous system Runx genes have been primarily linked to the peripheral nervous system [8] as well as olfactory bulb neurogenesis [9] and the differentiation of olfactory ensheathing glia [10]. In embryonic olfactory neural progenitor cells and in cortical progenitor cells, which are presumably most relevant to our study, Runx1 was also shown to promote cell proliferation while promoting spontaneous neuronal differentiation [9]. A conflicting role of Runx1 was reported in DRG progenitor cells, where Runx1 was inferred to be inhibitory to cell proliferation [11] and lead to neurite formation [12]. Therefore, it appears that the role of Runx1 in proliferation and differentiation varies depending on cell types. There has been no detailed information, however, on Runx functions in development of the central nervous system and adult neurogenesis in particular, except for the fact that in mice traumatic brain injury induced Tgfb and Runx1 expression in the neurogenic zones of the adult brain [13]. The expression of Runx2 in gliomas [14] and the fact that Runx1 was among the top 30 genes that have a genome-wide dosage effect in Down syndrome [15] might hint at complex functions of Runx genes in oncology and beyond and provide a link to neural stem cell biology.

Based on these data from the literature we hypothesized that Runx1 might be a candidate gene relevant for the switch between proliferation and neuronal differentiation in stem cells from the adult hippocampus as well. We used monolayer cultures from the hippocampus of adult mice [16] to investigate cell-autonomous effects of Runx1 on precursor cell function.

## Material and methods

### Animals and ethics statement

C57BL6/J mice (Charles River, Sulzfeld, Germany) with an age of 7–9 weeks were used to generate adult hippocampal neural precursor cells (NPCs).

This study was carried out in accordance with the rules of the German law on animal protection (TSchG) and the relevant guideline 2010/63/EU by the European Union.

For the qPCR studies, 10 week old C57BL/6JRj mice (Janvier) were housed singly in standard polycarbonate cages with or without a running wheel (150 mm diameter, TSE Systems, Germany). Hippocampal tissue used here was from the same animals as a previous study [17]. The protocol was approved by the internal Committee on the Ethics of Animal Experiments of the TU Dresden and the responsible authority Landesdirektion Sachsen (Permit number: 24–9168.11-1/2009-42).

### Isolation and cultivation of adult hippocampal precursor cells

The isolation and cultivation of adult hippocampal NPCs were performed as described previously [16,18,19]. Briefly, C57BL6/J mice with an age of 7~9 weeks were killed by cervical dislocation, and their hippocampal tissues were quickly isolated and minced in a petri dish with a scalpel blade. The minced hippocampi from 8~10 brains of C57BL6/J mice with an age of 7~9 weeks were digested in DMEM/F-12 medium containing papain (2.5 U/ml), dispase (1 U/ml), and deoxyribonuclease (250 U/ml) for 30~40 minutes at 37°C. The digested tissues were washed twice with Hank’s buffered saline solution (HBSS), resuspended in phosphate-buffered saline (PBS) containing 22% Percoll, and centrifuged at 450 x *g* for 15 minutes. The pellet fraction enriched with NPCs was then seeded in a culture plate, which was sequentially pre-coated with poly-D-lysine (PDL, 10 μg/ml) and laminin (5 μg/ml). NPCs were maintained and expanded in Neurobasal A medium supplemented with 2% B27, 1X GlutaMAX, 20 ng/ml human basic fibroblast growth factor (bFGF, PeproTech), and 20 ng/ml epidermal growth factor (EGF, PeproTech). For some experiments, NPCs were treated with TGF-β1 (PeproTech) for the periods indicated in the text and figure.

For induction of differentiation, NPCs were placed in the above medium but containing 10 ng/ml each of bFGF and EGF for 2 days and then maintained for additional 1~5 days in the medium containing 5~10 ng/ml bFGF before analysis.

### RNA isolation and RT-(q)PCR

**Fig 1:**
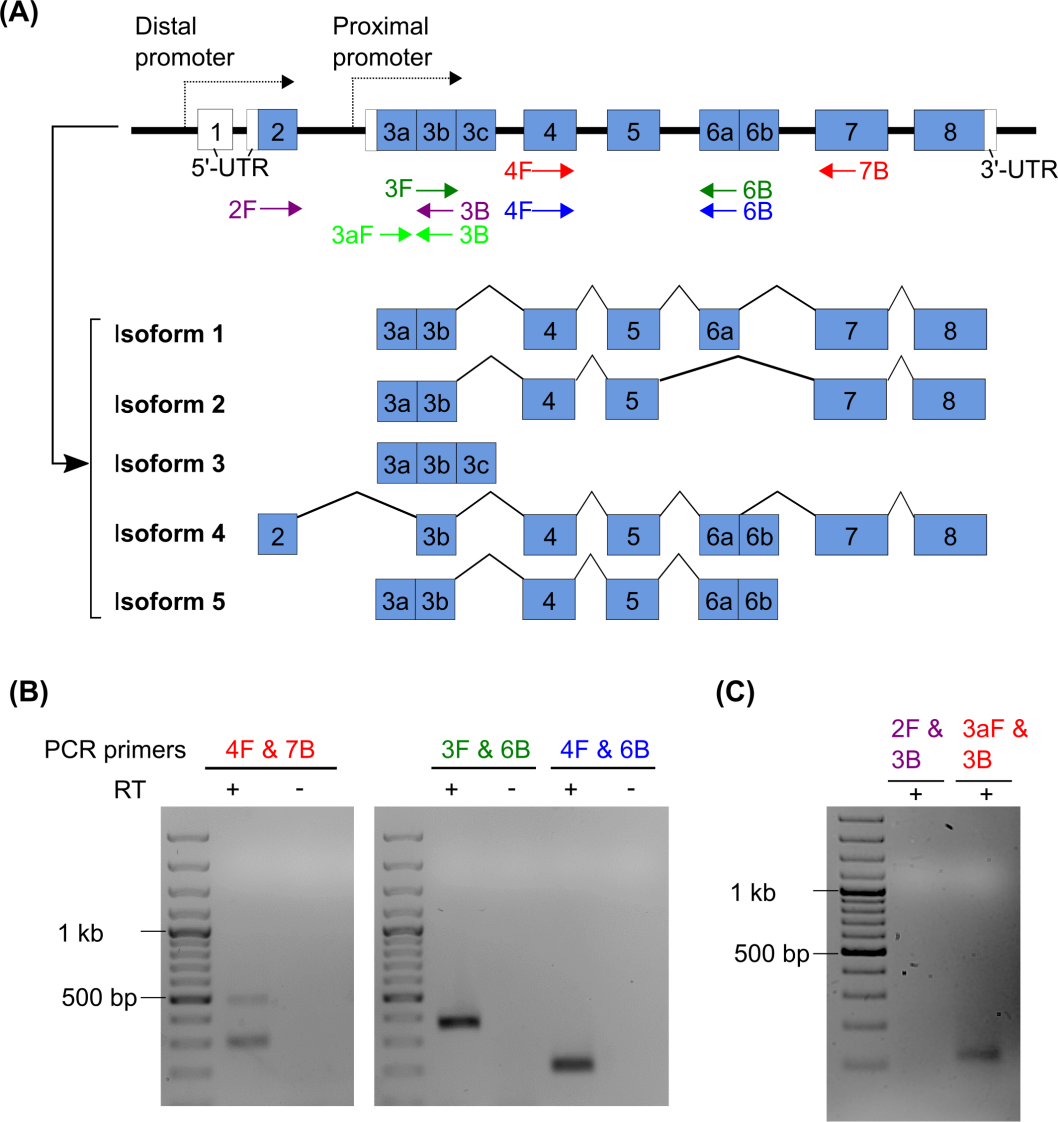
mRNA detection of different mouse Runx1 splicing variants in adult hippocampal NPCs. **(A)** Schematic diagrams illustrating the genomic structure and five known splicing variants of mouse Runx1. Those splicing variants encode Isoforms 1 ~ 5 (Uniprot identifiers: Q03347-1, Q03347-2, Q03347-3, Q03347-4, and Q03347-5). Positions of primers used in the subsequent RT-PCR experiments (mRunx1_2F, 3F, 3aF, 4F, 3B, 6B, and 7B) are also illustrated. **(B)** RT-PCR to detect transcripts encoding Isoforms 1 and 2. The primer pair of 4F & 7B detected two bands at ~500 and ~300 bps, which were expected from amplification of Isoform 1 and 2 transcripts, respectively. The pair of 3F & 6B or 4F & 6B, which targeted both the isoforms, amplified an expected single band of ~384 or ~223 bp, respectively. Isolated total RNA without reverse transcription (RT) was used as a negative control. **(C)** RT-PCR to detect P1- and P2- derived transcripts with the indicated primer pairs. Only the P2-derived transcript was detected. RT-PCR designed to specifically detect Isoform 3 or 5 did not yield any amplicons (data not shown). See S1 Table for the specific sequences of each primer used in this study.

Total RNA was extracted from sub-confluent monolayer culture of NPCs plated in a 6-well plate using the RNeasy Mini kit (Qiagen). The isolated RNA was then reverse-transcribed to cDNA using oligo(dT) primers and SuperScript II Reverse Transcriptase (Invitrogen) and was subjected to either conventional PCR or quantitative PCR (qPCR). PCR SuperMix (Invitrogen) and SYBR green PCR mix (Qiagen) were used to amplify target sequences for conventional and quantitative PCR, respectively. Relative levels of target transcripts were quantified using the relative standard curve method using β-actin as a reference gene. Primer pairs and sequences used in this study are summarized in S1 Table and also illustrated in Fig 1.

For the qPCR, cDNA from total mRNA isolated from dissected hippocampus was generated and qPCR performed as above. Primers used were “Runx1-q-all-fwd”, “Runx1-q-all-rev”, “Actb-fwd” and “Actb-rev” (S1 Table).

### Plasmid generation and nucleofection

DNA sequence encoding isoform b (Runx1b, Uniprot identifier Q01196-1) of human Runx1 was kindly provided by Dr. Carol Stocking (Heinrich-Pette-Institute, Leibniz-Institute for Experimental Virology, Hamburg, Germany) and was subcloned into pCMS-EGFP vector (Clontech). DNA sequence encoding isoform c (Runx1c, Uniprot identifier Q01196-3) of human Runx was obtained from Origene and similarly subcloned into pCMS-EGFP vector. The generated expression plasmids were purified using the EndoFree Plasmid Maxi kit (Qiagen) before being used for nucleofection.

Transfection of NPCs with the Runx1 overexpressing plasmids or pmaxGFP control plasmid (Lonza) was performed using the Amaxa Nucleofector Technology and mouse NSC nucleofector kit (Lonza) according to the manufacturer’s instruction. Briefly, 4 x 10^6^ NPCs were harvested after accutase treatment, resuspended in 100 μl nucleofector solution containing 7.5μg plasmid, and subjected to nucleofection in a supplied certified cuvette using the Nucleofector Program A-033. The transfected cells were then transferred to a separate tube, pelleted by centrifugation, resuspended in 1 ml culture medium, and plated into a 24-well plate at a density of 5 x 10^4^ cells/well.

### Western blot

NPCs were lysed directly in a culture plate with RIPA buffer (0.1% SDS, 0.25% sodium deoxycholate, 1% NP-40, 150 mM NaCl, 1 mM EDTA, 50 mM Tris-HCl pH 7.5) supplemented with protease inhibitor cocktail (Roche). Protein concentrations of NPC lysates were determined using the Dc Protein Assay kit (Bio-rad). Proteins were separated by SDS-PAGE in 10% polyacrylamide gels and transferred to nitrocellulose membrane. Blotted membrane was blocked with 5% milk and probed with rabbit polyclonal anti-Runx1 antibody (Millipore, #PC284, 1:2000) or mouse anti-GAPDH antibody (Millipore, #MAB374, 1:5000). Horseradish peroxidase (HRP)-conjugated secondary antibodies were then applied and detected with ECL Western Blotting Substrate (Pierce).

### Immunocytochemistry

NPCs plated on PDL/laminin-coated coverslips were fixed with ice-cold 4% paraformaldehyde (PFA) for 10 minutes. Cells were blocked for 1 hour in PBS containing 10% donkey serum and 0.2% Triton X-100 and then incubated overnight at 4°C with one or more of the following primary antibodies: mouse anti-βIII-tubulin (1:2000, Promega, #G7121, Clone Tuj1), mouse anti-MAP2 (1:2000, Sigma-Aldrich, #M1406), rabbit anti-GFP (1:1000, Invitrogen, # A-11122), and rat anti-BrdU (1:500, AbD Serotec, #OBT0030). On the next day, cells were incubated with Cy3- or DyLight488-conjugated secondary antibodies for 2 hours at room temperature. Nuclei were stained with Hoechst or DAPI, and the cells were mounted onto glass slides for microscopic evaluation.

For BrdU incorporation assay, NPCs were pretreated with 10 μM BrdU for 2 hours before fixation. Cells were sequentially washed with Tris-buffered saline (TBS) and 0.9% saline and then treated with 1M HCl for 30 minutes before going through the above staining procedures.

### Annexin V staining

NPCs transfected with Runx1b, Runx1c, or empty vector (pCMS-EGFP) were incubated with Annexin V-Cy3 (Abcam) for 5 minutes prior to PFA fixation. Cy3 and GFP fluorescence was then detected by fluorescence microscope.

### Sholl analysis

NPCs transfected with Runx1b, Runx1c, or empty vector (pCMS-EGFP) were initially cultured in the proliferation medium for 2 days and then for additional 5 days in the differentiation medium containing 5 ng/ml FGF2 but no EGF. The differentiated culture was stained with anti-MAP2 (2a+2b) antibody as described above. The length and complexity of MAP2+ processes in GFP+ cells were evaluated by Sholl Analysis with the application of ImageJ software (NIH) and the plugin developed by the Ghosh Lab (http://labs.biology.ucsd.edu/ghosh/software); a series of concentric circles spaced at 5 μm intervals were generated around the soma of a MAP2- and GFP- double positive cell, and the number of intersections (Y-axis) between the MAP2+ neurites and a circle placed at a given distance from the soma (X-axis) was counted. Error bars: SEM. n = 227~272 cells/group.

### Systems genetics

Genetic and phenotypic data on BXD recombinant inbred strains of mice are publicly available at www.genenetwork.org. Residuals of Ki67-positive cells as measure for precursor cell proliferation in the dentate gyrus have been deposited in the data base as part of our 2006 study on natural variation of adult neurogenesis [1] under trait ID 10795. We used the inbuilt functions of Genenetwork to establish the correlation between phenotypic values for proliferation with data on gene transcription in the hippocampus of the same strains. Hippocampal transcriptomics data for 71 BXD strains (plus other mouse strains) had been generated as part of a consortial effort in 2009 [20]. We used the data set “Hippocampus Consortium M430v2 (Jun06) PDNN for further analysis. The resulting list of 1000 genes whose expression correlated with Runx1 expression was submitted to WebGestalt GSAT (http://www.webgestalt.org) for functional enrichment analysis.

## Results

### Hippocampal precursor cells express Runx1 isoforms 1 and 2

We have developed a monolayer culture of adult hippocampal NPCs from mice [16,18], which maintain the precursor cells as a homogenous population over many passages and, based on the expression of Nestin and Sox2, have a purity of more than 95% [19]. To examine if Runx1 is expressed in cultured hippocampal precursor cells, we performed RT-PCR using cDNA from proliferating cells as a template and observed a clear expression of Runx1 mRNA (Fig 1). For mouse Runx1 five splice variants are known (Fig 1A). We designed primer pairs to specifically distinguish their expression and found that isoforms 1 and 2 were expressed, whereas no transcript of isoforms 3 to 5 was detectable. Runx1 has two promoters, the distal P1 and the proximal P2 promoter [21], and this dual promoter structure has been highly conserved throughout evolution. Using several primer pairs we found that Runx1 expression in the precursor cells exclusively originated from the P2 promoter, which also drives the expression of isoform 1 and 2. The murine isoform 1 corresponds to the human isoforms b (hereafter Runx1b). The murine isoform 5 (which was not expressed in our cells and corresponds to human isoform 1c) lacks the transactivation domain in exon 7. In later experiments we have overexpressed human isoform c (Runx1c) as a putative dominant negative factor regulator of Runx1 effects.

### Runx1 is down-regulated with neuronal differentiation and up-regulated by TGF-β1

**Fig 2:**
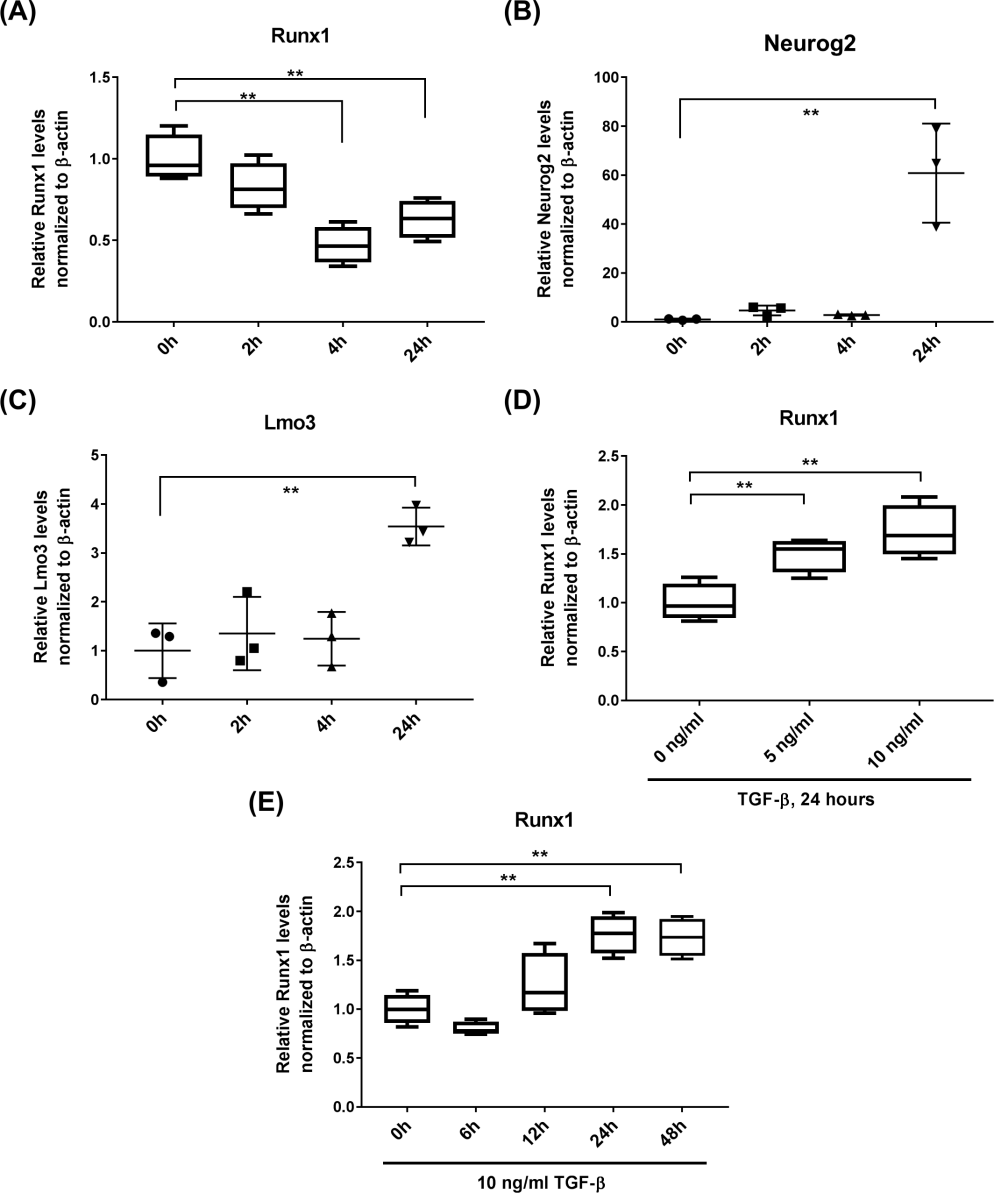
qRT-PCR to characterize a change in Runx1 expression in NPCs upon induction of differentiation. Isolated NPCs were initially expanded and maintained in a culture medium containing EGF and bFGF (10 ng/ml each). Differentiation was induced by removing EGF from the culture medium, and changes in the expression levels of Runx1 **(A)** as well as Neurog2 **(B)** and Lmo3 **(C)**, neuronal markers, were evaluated in the lysates collected at different time points after the removal of EGF. The primer pair of mRunx1_5F and mRunx1_6B was used for amplification of Runx1. See S1 Table for the specific sequences of each primer used in this study. **(D)** qRT-PCR to evaluate a change in Runx1 expression in NPCs upon treatment with TGF-β. Relative Runx1 mRNA levels in NPCs treated with 5 or 10 ng/ml TGF-β1 for 24 hours. **(E)** Relative Runx1 mRNA levels in NPCs treated with 10 ng/ml TGF-β1 for the indicated periods Error bars: standard deviation. n = 3~4/group. **: <0.01. Statistics: One-way ANOVA followed by Holm-Sidak’s multiple comparisons test.

Upon withdrawal of EGF in the cultures, Runx1 was down-regulated within hours, while marker genes for the neuronal lineage, Neurog2 and Lmo3 were up-regulated (Fig 2), suggesting that Runx1 is associated with the proliferative precursor cell state. Runx1 is known to be regulated by TGF-β1 [7]. In order to examine the question, whether Runx1 is regulated in the hippocampal precursor cells, cultures under proliferation conditions were treated with 5 or 10 ng/ml of TGF-β1. Under these conditions, we saw a dose-dependent increase in Runx1 expression (Fig 2). Taken together, these data indicated not only that adult neural stem cell express Runx1, as do stem cells in the other mentioned compartments, but also that this expression is embedded in similar regulatory pathways.

### Overexpression of Runx1 increases differentiation and survival, not proliferation

**Fig 3:**
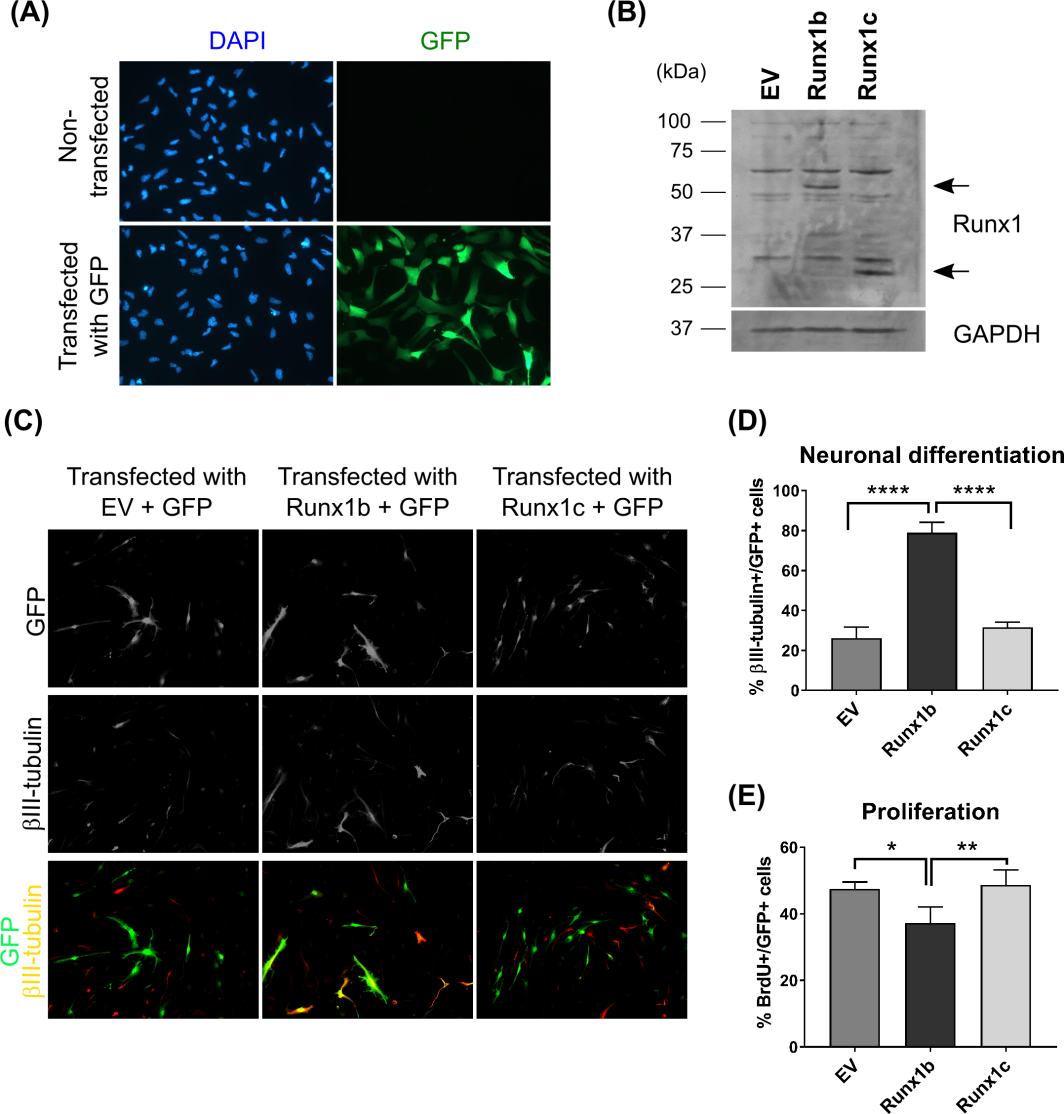
Overexpression of human Runx1 in proliferating NPCs using the Amaxa nucleofection system. **(A)** Assessment of transfection efficiency by detecting GFP fluorescence upon transfection with a control GFP plasmid (pmaxGFP). **(B)** Detection of overexpressed human Runx1 by Western blotting. Overexpressed human Runx1b and Runx1c, which correspond to murine Isoforms 1 and 5, respectively, were detected with rabbit polyclonal anti-Runx antibody. NPCs transfected with empty vector (EV, pCMS-EGFP vector) were loaded as a negative control. **(C)** Immunofluorescence images for GFP (upper panels) and βIII-tubulin (lower panels) from the three experimental groups. Cells showing both GFP and βIII-tubulin fluorescence were highlighted with arrows. **(D)** Proportion of βIII-tubulin- and GFP- double positive cells over total GFP-positive cells. n = 3/group. **(E)** Proportion of BrdU- and GFP- double positive cells over total GFP-positive cells. Transfected NPCs were cultured in the proliferation medium and subjected to BrdU incorporation assay: Cells were incubated with 10μM BrdU for 2 hours before fixation. The fixed cells were co-stained with anti-BrdU and anti-GFP antibodies. n = 4/group. Error bars: standard deviation. *: p<0.05. **: p<0.01. ****: p<0.0001. Statistics: One-way ANOVA followed by Holm-Sidak’s multiple comparisons test.

To evaluate the functional role of Runx1 in the cultured precursor cells, we used the Amaxa nucleofection system. We found 60–90% of the cells to be transfected (Fig 3). We subcloned human Runx1b and human Runx1c, which lacks the C-terminal transactivation domain, into a mammalian expression vector pCMS-EGFP (Clontech), which co-expresses eGFP. After transfection, GFP signal and Western blot confirmed the expression of both isoforms in the precursor cells (Fig 3).

**Fig 4:**
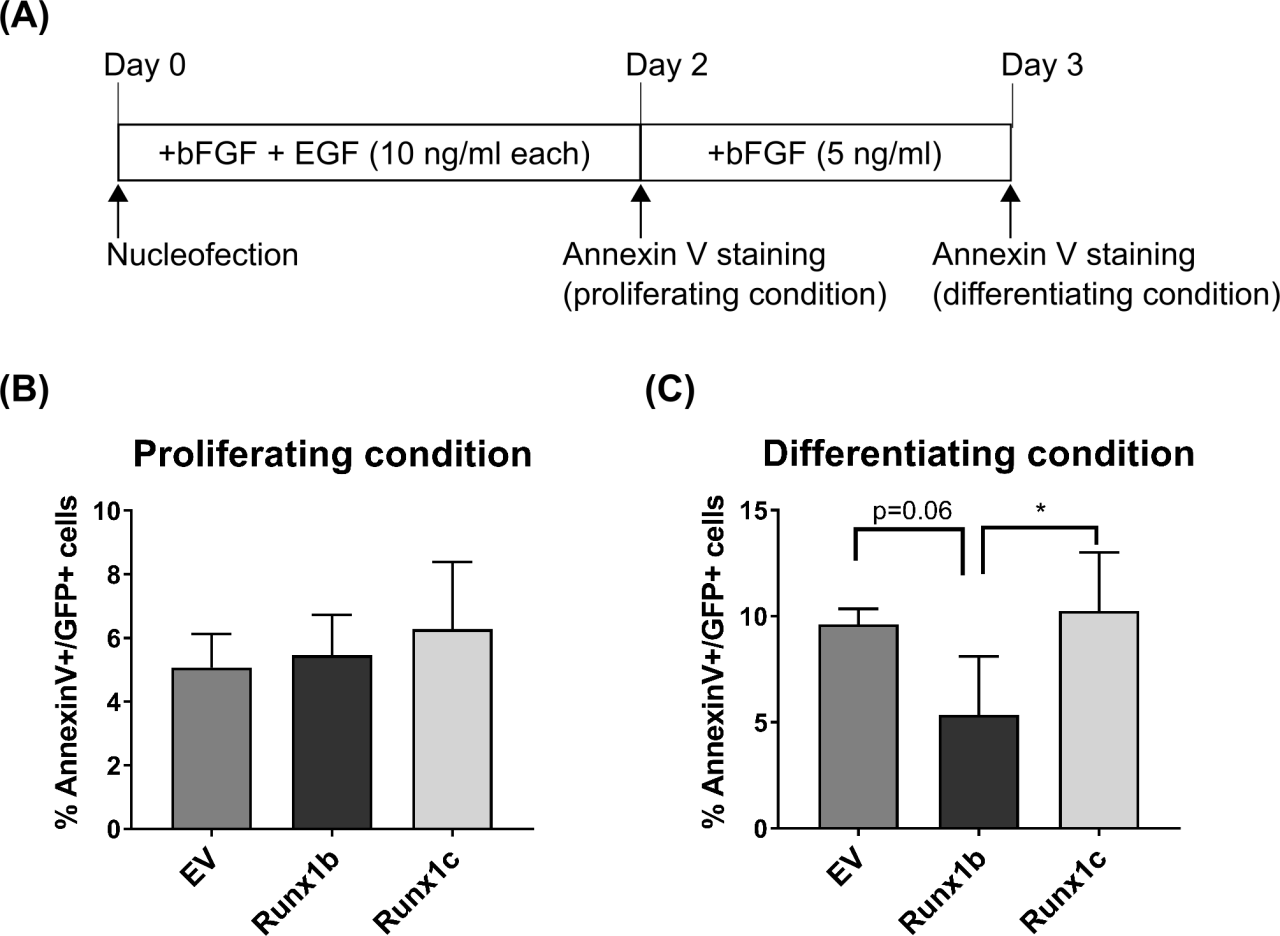
Effect of Runx1 overexpression on NPC survival in proliferating and differentiating conditions. **(A)** Schematic illustration of the experiment. Annexin V staining was performed on transfected NPCs either after 2 days of cultivation in the proliferation medium containing bFGF and EGF or after additional 1 day incubation in the differentiation medium not containing EGF. **(B and C)** Proportion of Annexin V- and GFP- double positive cells over total GFP-positive cells determined in the proliferating condition (B) or differentiating condition (C).

However, in contrast to data on various hematopoietic cells, we did not observe a pro-proliferative effect of Runx1 in our precursor cells. Nevertheless, overexpression of Runx1b, but not Runx1c, resulted in the generation of more neuronal cells under differentiation conditions (Fig 4). This result suggests that Runx1 may in fact have a role in promoting NPCs toward the differentiation into neurons.

Overexpression of Runx1 did not affect apoptosis as assessed with the Annexin V assay under proliferation condition but reduced programmed cell death under differentiating conditions (at 3 days after transfection and 1 day after withdrawal of EGF; Fig 4).

**Fig 5:**
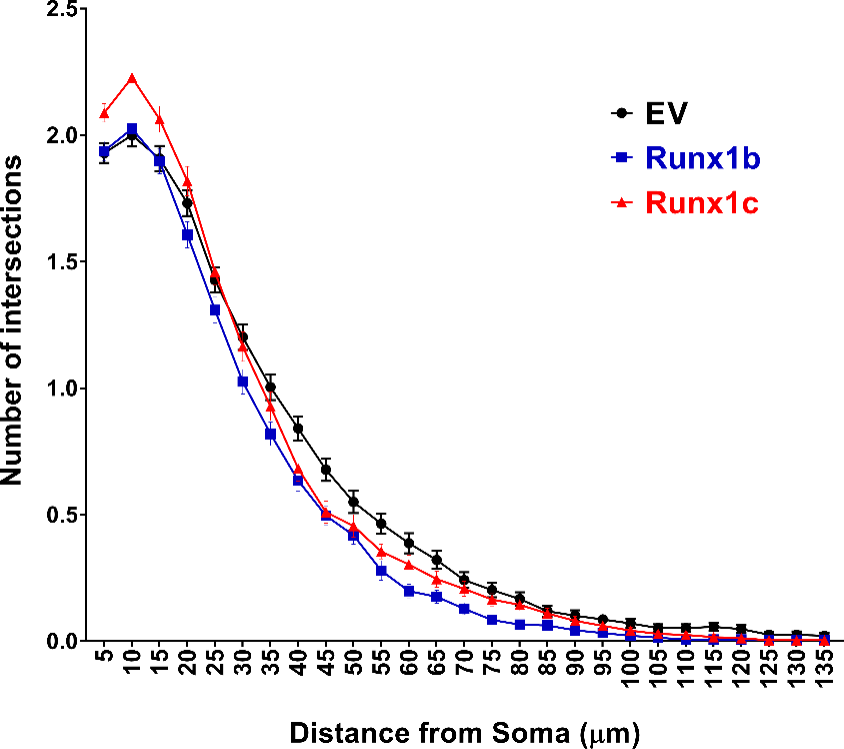
Sholl analysis to characterize effect of Runx1 overexpression on morphology of differentiated neurons. The length and complexity of MAP2+ processes in GFP+ cells were evaluated by Sholl Analysis (See Materials and methods for details).

A Sholl analysis in vitro revealed that overexpression of wildtype Runx1 in vitro slightly reduced the number of branch points in the neurites between 20 and 90μm from the soma, whereas the dominant negative isoform increased branches at short distances (5 to 20 μm) from the soma (Fig 5). The effect was very small, however.

### No exercise-induced induction of Runx1 in vivo

**Fig 6:**
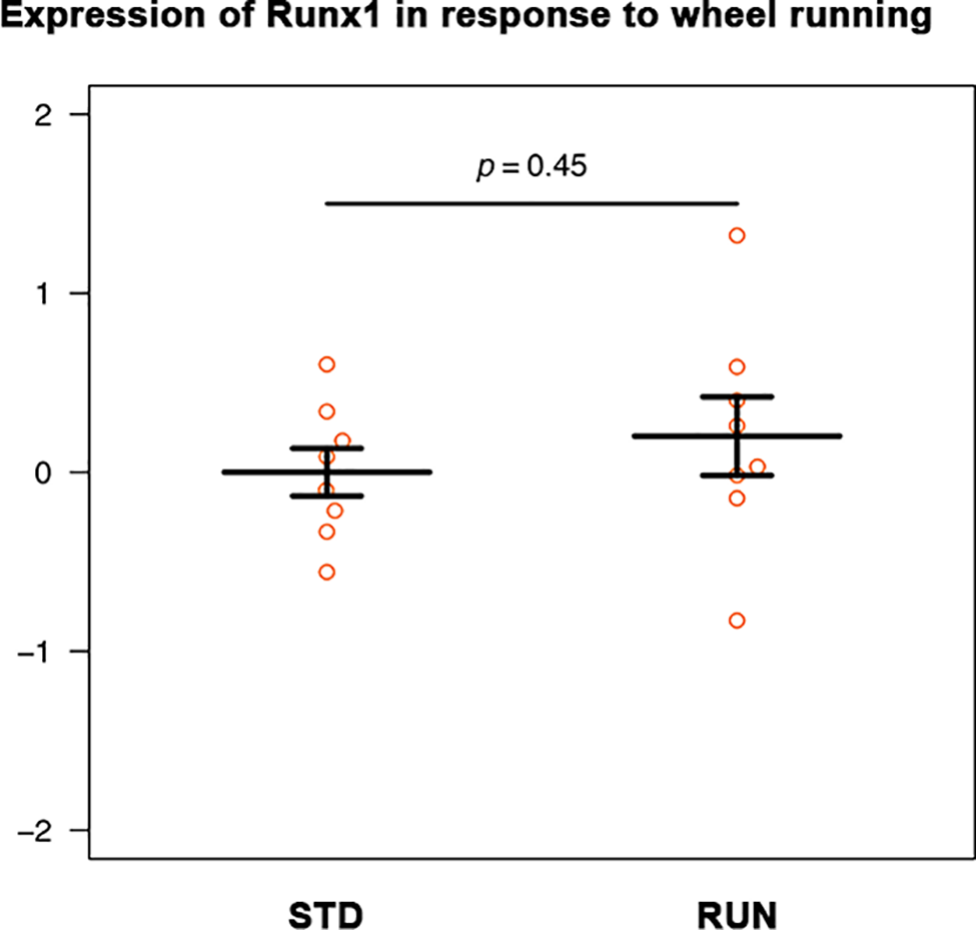
No measurable increase in Runx1 expression in the hippocampus in response to 4 days of voluntary wheel running, a strong pro-neurogenic stimulus (qPCR).

After unpublished microarray data had suggested that Runx1 might be up-regulated in the hippocampus after exercise, we performed a qPCR analysis in microdissected samples after 4 days of exercise. This is a duration with a strong effect on adult neurogenesis [17]. For Runx1 expression, the variance was high, and the mean difference was not statistically significant (Fig 6; Welch t test, t = 0.78, df = 11.53, p-value = 0.45).

### A systems genetics perspective on Runx1 in the murine hippocampus

To independently support a potential role of Runx1 in adult hippocampal neurogenesis in vivo we turned to our extensive analysis of adult hippocampal neurogenesis in the BXD panel of recombinant inbred strains of mice [1]. In essence, RI strains are inbred strains derived from brother-sister matings in the F2 generation of an initial cross between two parental strains, in this case C57BL/6J and DBA/2J. In this data set we could show that adult neurogenesis is a highly polygenic trait with small effect sizes in explaining trait variance attributable to many individual genetic loci [1]. We correlated precursor cell proliferation levels in the 24 strains of this data set (Genenetwork ID 10795) with hippocampal transcriptome data obtained in these strains [20]. Runx1 expression ranked 59^th^ among the genes that correlated with proliferation (r = 0.631; p = 0.00066). When we analyzed the top 1000 genes correlating with Runx1 expression in the entire transcriptomic data set (across 71 BXD strains) with the help of WebGestalt [22], we found for “biological process” among the 5 GO category at the lowest level “positive regulation of neural precursor cell proliferation” (GO:2000179) and “neuron differentiation” (GO:0030182). Correlating transcripts in these categories included Sox1, Sox5, Hif1a, Ephb2, Smo, Shh, Ngfr, Neurog3, Shank1, Sema6c, Egfr and Igf1.

## Discussion

We report here that the known “stem cell” gene Runx1 exerts a pro-neurogenic function in neural precursor cells in vitro, while in vivo data remained inconclusive. Given the interest of our group in activity-dependent regulation of adult neurogenesis, Runx1 had been a plausible candidate for a relevant gene in this context. The human homolog RUNX1 has also been found to be up-regulated by endurance training in skeletal muscle [23], where it is known that Runx1, together with, for example, MyoD, MuRF1and myostatin controls muscle mass. There were no data on brain as yet. After suggestive pilot data in a micro-array experiment, we could not verify, however, that Runx1 would be up-regulated in the hippocampus after running.

In muscle, Runx1 is also very lowly expressed under physiological conditions but required to maintain muscle mass in the case of denervation [24], suggesting an involvement in maintenance functions. In many systems, including adult neurogenesis, maintenance is secured by stem cell activity in the system. As exercise not only acutely stimulates precursor cell proliferation but also has more lasting effects on later stages of neurogenesis and the maintenance of the precursor cell pool over prolonged periods of time, this link also justified a hypothesis relating Runx1 to adult hippocampal neurogenesis.

Expression of Runx1 in neural precursor cells of the adult brain has been reported after traumatic brain injury. In that context, Runx1 expression was strongest in microglial cells of the neurogenic regions, consistent with the key function of Runx1 in the hematopoetic system and the inducible regulation in adult neurogenesis [13]. A follow-up study by Logan et al. [25] investigated Runx1 in neurosphere cultures from the subventricular zone and reported a role in proliferation and differentiation, but not survival. In contrast, we focused on the hippocampus and used monolayer cultures. We also found an effect on cell survival. The two neurogenic regions are developmentally distinct, produce different types of neurons, are characterized by different sets of transcription factors, and are regulated by different mechanisms (see Table 8–2 and related text in Ref. [26], p. 287). Consequently, conclusions cannot be easily translated from one regions to the other. In addition, neurospheres and monolayers have different properties. In monolayers, the precursor cells are more isolated and the culture are less heterogeneous.

Runx1 is a transcription factor that is crucial for the differentiation of hematopoietic stem or progenitor cells into specific hematopoietic lineages in adult hematopoesis [27]. Runx1 protein is characterized by the presence of a DNA-binding domain called Runt domain at its N-terminus and a transcription activation domain at its C-terminus [28]. In association with other cofactors, Runx1 is known to bind to a conserved DNA motif at gene regulatory regions and regulate the expression of hematopoesis-associated genes. Nevertheless, is was shown that Runx1 is dispensable for the function of hematopoetic stem cells in the adult [29]. This might be in line with our findings with dominant negative Runx1. Although multiple lines of evidence suggest Runx1 acts as a pro-proliferative factors in hematopoietic stem cells and hair follicle stem cells [4], a contradicting anti-proliferative and pro-neurogenic role of Runx1 was suggested in developing DRGs [28]. Our finding from the overexpression study is in line with the latter finding, and it could be that Runx1 plays a similar role in developing DRGs and NPCs [29]. It remains to be determined whether no obvious effect of Runx1c in our study was due to its insufficient expression to compete with endogenous mouse Runx1.

The regulatory mechanism of Runx1 expression is still rather poorly understood [30]. The expression of Runx1 is governed by the two distinct promoters; a distal P1 promoter and a proximal P2 promoter [31]. The alternative usage of promoters results in distinct Runx1 transcripts with different 5’-UTR and N-terminus coding sequences, providing a complex regulatory mechanism of Runx1 functions. It remained to be evaluated whether the promoter usage is spatially and temporarily regulated in adult animals and whether physiological or pathological stimulation affects the promoter usage. While specific transcription factors that mediate the transcription of Runx1 have not been characterized, some extrinsic factors were reported to upregulate Runx1. Those factors include TGF-β [32], Notch1 [33], and luteinizing hormone [34].

By and large, our results fit the picture drawn by the literature that depending on lineage, Runx1, can be involved in different regulatory mechanisms at the stem cell level. We can now add adult hippocampal precursor cells to the list of cells responsive to Runx1. But we have also seen that the observed effects are limited and our in vivo data have, despite considerable experimental effort, remained inconclusive.

Within the wider context Runx1 and such genes of low effect size would not be irrelevant and their involvement in the process of interest is indeed substantial but their role is not central and mandatory. Our small systems genetics analysis confirmed that Runx1 expression in the murine hippocampus in vivo correlates with a number of known neurogenesis-related genes, including Shh, Egfr, Neurog3, Igf1 and others.

It might well be that with experimental paradigms such as ours (and a candidate gene like Runx1) we start thus looking at regulatory events at a secondary level. Highly polygenic traits such as adult hippocampal neurogenesis must involve many such relevant genes of very limited effect size [35].

From a systems biology perspective the existence of such modulators makes perfect sense, but the problem remains how to integrate such knowledge into a bigger picture of how regulation (of adult neurogenesis) occurs. The present study shows that even a detailed analysis of individual candidates with high face value validity reaches a limit.

## Supporting information

S1 Table Primer pairs used in the current study. (DOCX)

S1 File Data obtained in this study. (ZIP)

## References

[1] Kempermann G, Chesler EJ, Lu L, Williams RW, Gage FH. Natural variation and genetic covariance in adult hippocampal neurogenesis. Proceedings of the National Academy of Sciences. 2006;103: 780–785. doi: 10.1073/pnas.0510291103 16407118 10.1073/pnas.0510291103 PMC1325968

[2] Kempermann G. Seven principles in the regulation of adult neurogenesis. Eur J Neurosci. Blackwell Publishing Ltd; 2011;33: 1018–1024. doi: 10.1111/j.1460-9568.2011.07599.x 21395844 10.1111/j.1460-9568.2011.07599.x

[3] Coffman JA. Runx transcription factors and the developmental balance between cell proliferation and differentiation. Cell Biol Int. 2003;27: 315–324. 12788047 10.1016/s1065-6995(03)00018-0

[4] Appleford PJ, Woollard A. RUNX genes find a niche in stem cell biology. J Cell Biochem. 2009;108: 14–21. doi: 10.1002/jcb.22249 19562739 10.1002/jcb.22249

[5] Yokomizo T, Ogawa M, Osato M, Kanno T, Yoshida H, Fujimoto T, et al Requirement of Runx1/AML1/PEBP2alphaB for the generation of haematopoietic cells from endothelial cells. Genes Cells. 2001;6: 13–23. 11168593 10.1046/j.1365-2443.2001.00393.x

[6] Kagoshima H, Nimmo R, Saad N, Tanaka J, Miwa Y, Mitani S, et al The C. elegans CBFbeta homologue BRO-1 interacts with the Runx factor, RNT-1, to promote stem cell proliferation and self-renewal. Development. The Company of Biologists Ltd; 2007;134: 3905–3915. doi: 10.1242/dev.008276 17933794 10.1242/dev.008276

[7] Ito Y, Miyazono K. RUNX transcription factors as key targets of TGF-beta superfamily signaling. Current Opinion in Genetics & Development. 2003;13: 43–47. 12573434 10.1016/s0959-437x(03)00007-8

[8] Wang JW, Stifani S. Roles of Runx Genes in Nervous System Development. Adv Exp Med Biol. Singapore: Springer Singapore; 2017;962: 103–116. doi: 10.1007/978-981-10-3233-2_8 28299654 10.1007/978-981-10-3233-2_8

[9] Theriault FM, Nuthall HN, Dong Z, Lo R, Barnabé-Heider F, Miller FD, et al Role for Runx1 in the proliferation and neuronal differentiation of selected progenitor cells in the mammalian nervous system. J Neurosci. 2005;25: 2050–2061. doi: 10.1523/JNEUROSCI.5108-04.2005 15728845 10.1523/JNEUROSCI.5108-04.2005 PMC6726063

[10] Murthy M, Bocking S, Verginelli F, Stifani S. Transcription factor Runx1 inhibits proliferation and promotes developmental maturation in a selected population of inner olfactory nerve layer olfactory ensheathing cells. Gene. 2014;540: 191–200. doi: 10.1016/j.gene.2014.02.038 24582971 10.1016/j.gene.2014.02.038

[11] Yoshikawa M, Senzaki K, Yokomizo T, Takahashi S, Ozaki S, Shiga T. Runx1 selectively regulates cell fate specification and axonal projections of dorsal root ganglion neurons. Dev Biol. 2007;303: 663–674. doi: 10.1016/j.ydbio.2006.12.007 17208218 10.1016/j.ydbio.2006.12.007

[12] Yoshikawa M, Masuda T, Kobayashi A, Senzaki K, Ozaki S, Aizawa S, et al Runx1 contributes to the functional switching of bone morphogenetic protein 4 (BMP4) from neurite outgrowth promoting to suppressing in dorsal root ganglion. Mol Cell Neurosci. 2016;72: 114–122. doi: 10.1016/j.mcn.2016.02.001 26892431 10.1016/j.mcn.2016.02.001

[13] Logan TT, Villapol S, Symes AJ. TGF-β superfamily gene expression and induction of the Runx1 transcription factor in adult neurogenic regions after brain injury. Zheng JC, editor. PLoS ONE. Public Library of Science; 2013;8: e59250 doi: 10.1371/journal.pone.0059250 23555640 10.1371/journal.pone.0059250 PMC3605457

[14] Vladimirova V, Waha A, Lückerath K, Pesheva P, Probstmeier R. Runx2 is expressed in human glioma cells and mediates the expression of galectin-3. J Neurosci Res. Wiley Subscription Services, Inc., A Wiley Company; 2008;86: 2450–2461. doi: 10.1002/jnr.21686 18438928 10.1002/jnr.21686

[15] Vilardell M, Rasche A, Thormann A, Maschke-Dutz E, Pérez-Jurado LA, Lehrach H, et al Meta-analysis of heterogeneous Down Syndrome data reveals consistent genome-wide dosage effects related to neurological processes. BMC Genomics. BioMed Central; 2011;12: 229 doi: 10.1186/1471-2164-12-229 21569303 10.1186/1471-2164-12-229 PMC3110572

[16] Babu H, Claasen J-H, Kannan S, Rünker AE, Palmer T, Kempermann G. A protocol for isolation and enriched monolayer cultivation of neural precursor cells from mouse dentate gyrus. Front Neurosci. 2011;5: 89 doi: 10.3389/fnins.2011.00089 21811434 10.3389/fnins.2011.00089 PMC3140691

[17] Overall RW, Walker TL, Leiter O, Lenke S, Ruhwald S, Kempermann G. Delayed and Transient Increase of Adult Hippocampal Neurogenesis by Physical Exercise in DBA/2 Mice. PLoS ONE. 2013;8: e83797 doi: 10.1371/journal.pone.0083797 24376750 10.1371/journal.pone.0083797 PMC3869944

[18] Babu H, Cheung G, Kettenmann H, Palmer TD, Kempermann G. Enriched monolayer precursor cell cultures from micro-dissected adult mouse dentate gyrus yield functional granule cell-like neurons. Chan-Ling T, editor. PLoS ONE. 2007;2: e388 doi: 10.1371/journal.pone.0000388 17460755 10.1371/journal.pone.0000388 PMC1849968

[19] Kannan S, Nicola Z, Overall RW, Ichwan M, Ramirez-Rodriguez G, N Grzyb A, et al Systems Genetics Analysis of a Recombinant Inbred Mouse Cell Culture Panel Reveals Wnt Pathway Member Lrp6 as a Regulator of Adult Hippocampal Precursor Cell Proliferation. Stem Cells. 2016;34: 674–684. doi: 10.1002/stem.2313 26840599 10.1002/stem.2313

[20] Overall RW, Kempermann G, Peirce J, Lu L, Goldowitz D, Gage FH, et al Genetics of the hippocampal transcriptome in mouse: a systematic survey and online neurogenomics resource. Front Neurosci. 2009;3: 55–10. doi: 10.3389/neuro.15.003.2009 20582282 10.3389/neuro.15.003.2009 PMC2858614

[21] Sroczynska P, Lancrin C, Kouskoff V, Lacaud G. The differential activities of Runx1 promoters define milestones during embryonic hematopoiesis. Blood. 2009;114: 5279–5289. doi: 10.1182/blood-2009-05-222307 19858498 10.1182/blood-2009-05-222307

[22] Wang J, Vasaikar S, Shi Z, Greer M, Zhang B. WebGestalt 2017: a more comprehensive, powerful, flexible and interactive gene set enrichment analysis toolkit. Nucleic Acids Res. 2017 doi: 10.1093/nar/gkx356 28472511 10.1093/nar/gkx356 PMC5570149

[23] Keller P, Vollaard NBJ, Gustafsson T, Gallagher IJ, Sundberg CJ, Rankinen T, et al A transcriptional map of the impact of endurance exercise training on skeletal muscle phenotype. J Appl Physiol. American Physiological Society; 2011;110: 46–59. doi: 10.1152/japplphysiol.00634.2010 20930125 10.1152/japplphysiol.00634.2010 PMC3253010

[24] Wang X, Blagden C, Fan J, Nowak SJ, Taniuchi I, Littman DR, et al Runx1 prevents wasting, myofibrillar disorganization, and autophagy of skeletal muscle. Genes & Development. Cold Spring Harbor Lab; 2005;19: 1715–1722. doi: 10.1101/gad.1318305 16024660 10.1101/gad.1318305 PMC1176009

[25] Logan TT, Rusnak M, Symes AJ. Runx1 promotes proliferation and neuronal differentiation in adult mouse neurosphere cultures. Stem Cell Res. Elsevier B.V; 2015;15: 554–564. doi: 10.1016/j.scr.2015.09.014 26473321 10.1016/j.scr.2015.09.014

[26] Kempermann G. Neurogenic and non-neurogenic regions. In: Kempermann G, editor. Adult Neurogenesis 2. 2nd ed. Oxford, New York; 2011.

[27] Kumano K, Kurokawa M. The role of Runx1/AML1 and Evi-1 in the regulation of hematopoietic stem cells. J Cell Physiol. Wiley Subscription Services, Inc., A Wiley Company; 2010;222: 282–285. doi: 10.1002/jcp.21953 19847803 10.1002/jcp.21953

[28] Cameron ER, Neil JC. The Runx genes: lineage-specific oncogenes and tumor suppressors. Oncogene. Nature Publishing Group; 2004;23: 4308–4314. doi: 10.1038/sj.onc.1207130 15156187 10.1038/sj.onc.1207130

[29] Ichikawa M, Asai T, Saito T, Seo S, Yamazaki I, Yamagata T, et al AML-1 is required for megakaryocytic maturation and lymphocytic differentiation, but not for maintenance of hematopoietic stem cells in adult hematopoiesis. Nat Med. Nature Publishing Group; 2004;10: 299–304. doi: 10.1038/nm997 14966519 10.1038/nm997

[30] Kobayashi A, Senzaki K, Ozaki S, Yoshikawa M, Shiga T. Runx1 promotes neuronal differentiation in dorsal root ganglion. Mol Cell Neurosci. 2012;49: 23–31. doi: 10.1016/j.mcn.2011.08.009 21906677 10.1016/j.mcn.2011.08.009

[31] Levanon D, Glusman G, Bangsow T, Ben-Asher E, Male DA, Avidan N, et al Architecture and anatomy of the genomic locus encoding the human leukemia-associated transcription factor RUNX1/AML1. Gene. 2001;262: 23–33. 11179664 10.1016/s0378-1119(00)00532-1

[32] Wildey GM, Howe PH. Runx1 is a co-activator with FOXO3 to mediate transforming growth factor beta (TGFbeta)-induced Bim transcription in hepatic cells. Journal of Biological Chemistry. American Society for Biochemistry and Molecular Biology; 2009;284: 20227–20239. doi: 10.1074/jbc.M109.027201 19494111 10.1074/jbc.M109.027201 PMC2740449

[33] Meier-Stiegen F, Schwanbeck R, Bernoth K, Martini S, Hieronymus T, Ruau D, et al Activated Notch1 target genes during embryonic cell differentiation depend on the cellular context and include lineage determinants and inhibitors. Stanton J-AL, editor. PLoS ONE. Public Library of Science; 2010;5: e11481 doi: 10.1371/journal.pone.0011481 20628604 10.1371/journal.pone.0011481 PMC2900208

[34] Jo M, Curry TE. Luteinizing hormone-induced RUNX1 regulates the expression of genes in granulosa cells of rat periovulatory follicles. Mol Endocrinol. 2006;20: 2156–2172. doi: 10.1210/me.2005-0512 16675540 10.1210/me.2005-0512 PMC1783681

[35] Boyle EA, Li YI, Pritchard JK. An Expanded View of Complex Traits: From Polygenic to Omnigenic. Cell. Elsevier; 2017;169: 1177–1186. doi: 10.1016/j.cell.2017.05.038 28622505 10.1016/j.cell.2017.05.038 PMC5536862

