# Supplementary material for: Transcription factor Runx1 is pro-neurogenic in adult hippocampal precursor cells
Source: PLoS One. 2018 Jan 11;13(1):e0190789. doi: 10.1371/journal.pone.0190789 (PMC5764282; doi:10.1371/journal.pone.0190789)
Supplement: S1 Table — (DOCX) [file pone.0190789.s001.docx]

**S1 Table.** **Primer pairs used in the current study**

| **Pair ID#** | **Application** | **Primer name** | **Sequence (5’ – 3’)** | **Expected amplicon size (bp)** |
| --- | --- | --- | --- | --- |
| 1 | RT-PCR | mRunx1_4F | GCCATGAAGAACCAGGTAGC | 502 and 310 (from isoform 1 and 2, respectively) |
|  |  | mRunx1_7B | GTTCTGCAGAGAGGCTGGTC |  |
| 2 | RT-PCR | mRunx1_3F | ACTTCCTCTGCTCCGTGCTA | 384 (from isoform 1) |
|  |  | mRunx1_6B | GCTCGGAAAAGGACAAACTC |  |
| 3 | RT-PCR | mRunx1_4F | GCCATGAAGAACCAGGTAGC | 223 (from isoform 1) |
|  |  | mRunx1_6B | GCTCGGAAAAGGACAAACTC |  |
| 4 | RT-PCR | mRunx1_2F | TTTCCTTCATATCCACAGTGCTT | 101 (from isoform 4) |
|  |  | mRunx1_3B | AGCGCCTCGCTCATCTTG |  |
| 5 | RT-PCR | mRunx1_3aF | ATGCGTATCCCCGTAGATGC | 86 (from isoform 1) |
|  |  | mRunx1_3B | AGCGCCTCGCTCATCTTG |  |
| 6 | qRT-PCR | mRunx1_5F | GCCATCAAAATCACAGTGGAC | 97 (from isoform 1) |
|  |  | mRunx1_6B | GCTCGGAAAAGGACAAACTC |  |
| 7 | qRT-PCR | mNeurog2_F | AGCCCTTCTCCACCTTCCT | 122 |
|  |  | mNeurog2_B | TGCCAGTAGTCCACGTCTGA |  |
| 8 | qRT-PCR | mLmo3_F | CTCAGTTCAGCCAGACACCA | 101 |
|  |  | mLmo3_B | TGCCAGTATTTGTCCAGTGC |  |
| 9 | qRT-PCR | mBactin_F | AAATCGTGCGTGACATCAAA | 178 |
|  |  | mBactin_B | AAGGAAGGCTGGAAAAGAGC |  |
| 10 | qRT-PCR | Runx1-q-all-fwd | CACTGGCGCTGCAAC | 171 (from all isoforms) |
|  |  | Runx1-q-all-rev | GAGGTCGTTGAATCT |  |
| 11 | qRT-PCR | Actb-fwd | ACCCGCGAGCACAGCTTC | 112 |
|  |  | Actb-rev | ACATGCCGGAGCCGTTGT |  |
